# Using repeatability of performance within and across contexts to validate measures of behavioral flexibility

**DOI:** 10.7717/peerj.15773

**Published:** 2023-08-17

**Authors:** Kelsey McCune, Aaron Blaisdell, Zoe Johnson-Ulrich, August Sevchik, Dieter Lukas, Maggie MacPherson, Benjamin Seitz, Corina J. Logan

**Affiliations:** 1 Institute for Social, Behavioral and Economic Research, University of California Santa Barbara, Santa Barbara, California, United States of America; 2 Department of Psychology, University of California Los Angeles, Los Angeles, California, United States of America; 3 Arizona State University, Tempe, Arizona, United States of America; 4 Max Planck Institute for Evolutionary Anthropology, Leipzig, Germany

**Keywords:** Behavioral flexibility, Repeatability, Construct validity, Animal cognition, Great-tailed grackles

## Abstract

Research into animal cognitive abilities is increasing quickly and often uses methods where behavioral performance on a task is assumed to represent variation in the underlying cognitive trait. However, because these methods rely on behavioral responses as a proxy for cognitive ability, it is important to validate that the task structure does, in fact, target the cognitive trait of interest rather than non-target cognitive, personality, or motivational traits (construct validity). Although it can be difficult, or impossible, to definitively assign performance to one cognitive trait, one way to validate that task structure is more likely to elicit performance based on the target cognitive trait is to assess the temporal and contextual repeatability of performance. In other words, individual performance is likely to represent an inherent trait when it is consistent across time and across similar or different tasks that theoretically test the same trait. Here, we assessed the temporal and contextual repeatability of performance on tasks intended to test the cognitive trait behavioral flexibility in great-tailed grackles (*Quiscalus mexicanus*). For temporal repeatability, we quantified the number of trials to form a color preference after each of multiple color reversals on a serial reversal learning task. For contextual repeatability, we then compared performance on the serial color reversal task to the latency to switch among solutions on each of two different multi-access boxes. We found that the number of trials to form a preference in reversal learning was repeatable across serial color reversals and the latency to switch a preference was repeatable across color reversal learning and the multi-access box contexts. This supports the idea that the reversal learning task structure elicits performance reflective of an inherent trait, and that reversal learning and solution switching on multi-access boxes similarly reflect the inherent trait of behavioral flexibility.

## Introduction

Research on the cognitive abilities of non-human animals is important for several reasons. By understanding animal cognitive abilities, we can clarify factors that influenced the evolution of human cognition, the mechanisms that relate cognition to ecological and evolutionary dynamics, or we can use the knowledge to facilitate more humane treatment of captive animals (Shettleworth, 2010). In the last 50 years, comparative psychologists and behavioral ecologists have led a surge in studies innovating methods for measuring cognitive traits in animals. As a result, we have come to understand cognition as the process of acquiring information, followed by storage, retrieval, and use of that information for guiding behavior (Shettleworth, 2010). Evidence now exists that various species possess cognitive abilities in both the physical (*e.g*., object permanence: Salwiczek et al., 2009; causal understanding: Taylor, Knaebe & Gray, 2012) and social domains (*e.g*., social learning: Hoppitt et al., 2012; transitive inference: MacLean, Merritt & Brannon, 2008).

Cognitive traits are not directly observable and nearly all methods to quantify cognition use behavioral performance as a proxy for cognitive ability. Consequently, it is important to evaluate the validity of the chosen methods for quantifying a cognitive trait. To better understand whether performance on a type of task is likely to reflect a target cognitive trait (*i.e*., that the method has construct validity), researchers can test for repeatability in individual performance within and across tasks (Völter et al., 2018). However, while many cognitive abilities have been tested, and various methods used, it is rare for one study to repeatedly test individuals with the same method or use multiple methods to test for a given cognitive ability. This could be problematic because cognitive traits are not directly observable, so nearly all methods use behavioral performance as a proxy for cognitive ability. Using only one method to measure a cognitive trait could be problematic because it is hard to discern whether non-target cognitive, personality, or motivational factors may be the cause of variation in performance on the task (Morand-Ferron, Cole & Quinn, 2016). For example, the success of pheasants on multiple similar and different problem-solving tasks was related to individual variation in persistence and motivation, rather than problem solving ability (van Horik & Madden, 2016). Additionally, performance on cognitive tasks can be affected by different learning styles, where individuals can vary in their perception of the salience of stimuli within a task, the impact of a reward (or non-reward) on future behavior, or the propensity to sample alternative stimuli (Rowe & Healy, 2014). By assessing the temporal and contextual repeatability of performance, researchers can quantify the proportion of variation in performance that is attributable to consistent individual differences likely to reflect the level of the cognitive trait relative to other ephemeral factors that affect individual performance (Cauchoix et al., 2018).

Behavioral flexibility, the ability to change behavior when circumstances change, is a general cognitive ability that likely affects interactions with both the social and physical environment (Bond, Kamil & Balda, 2007). Although by definition behavioral flexibility incorporates plasticity in behavior through learning, there is also evidence that the ability to change behavior could be an inherent trait that varies among individuals and species. For example, the pinyon jay-a highly social species of corvid-made fewer errors in a serial reversal learning task than the more asocial Clark’s nutcracker or Woodhouse’s scrub-jay, but all three species exhibited similar learning curves over successive reversals (Bond, Kamil & Balda, 2007). This indicates that the three species differed in the level of the inherent ability, but were similar in the plasticity of performance through learning. Behavioral flexibility could be measured using a variety of methods (Mikhalevich, Powell & Logan, 2017), but the most popular method is reversal learning (Bond, Kamil & Balda, 2007) where behavioral flexibility is quantified as the speed that individuals are able to switch a learned preference. However, to our knowledge, no studies have assessed the construct validity of this task by comparing performance of individuals over time and across different tasks that are predicted to require flexible behavior.

In the wild, this ability to change behavior when circumstances change is expected to result in individuals and species that adapt quickly to novelty by showing a high rate of foraging innovations. For example, cross-taxon correlational studies found that species that were “behaviorally flexible”, in that there were many documented foraging innovations, were also more likely to become invasive when introduced to novel habitats (Sol, Timmermans & Lefebvre, 2002). The ability to innovate solutions to novel problems can also be more directly quantified using a multi-access or puzzle box task, where the subject must use new behavior patterns to solve the task to get food. While it is generally assumed that foraging innovation rate corresponds to the cognitive ability behavioral flexibility (Sol, Timmermans & Lefebvre, 2002), few studies compare innovation performance and solution switching (a measure of flexibility) on a multi-access box task to performance on a different behavioral flexibility task like reversal learning.

**Figure 1:**
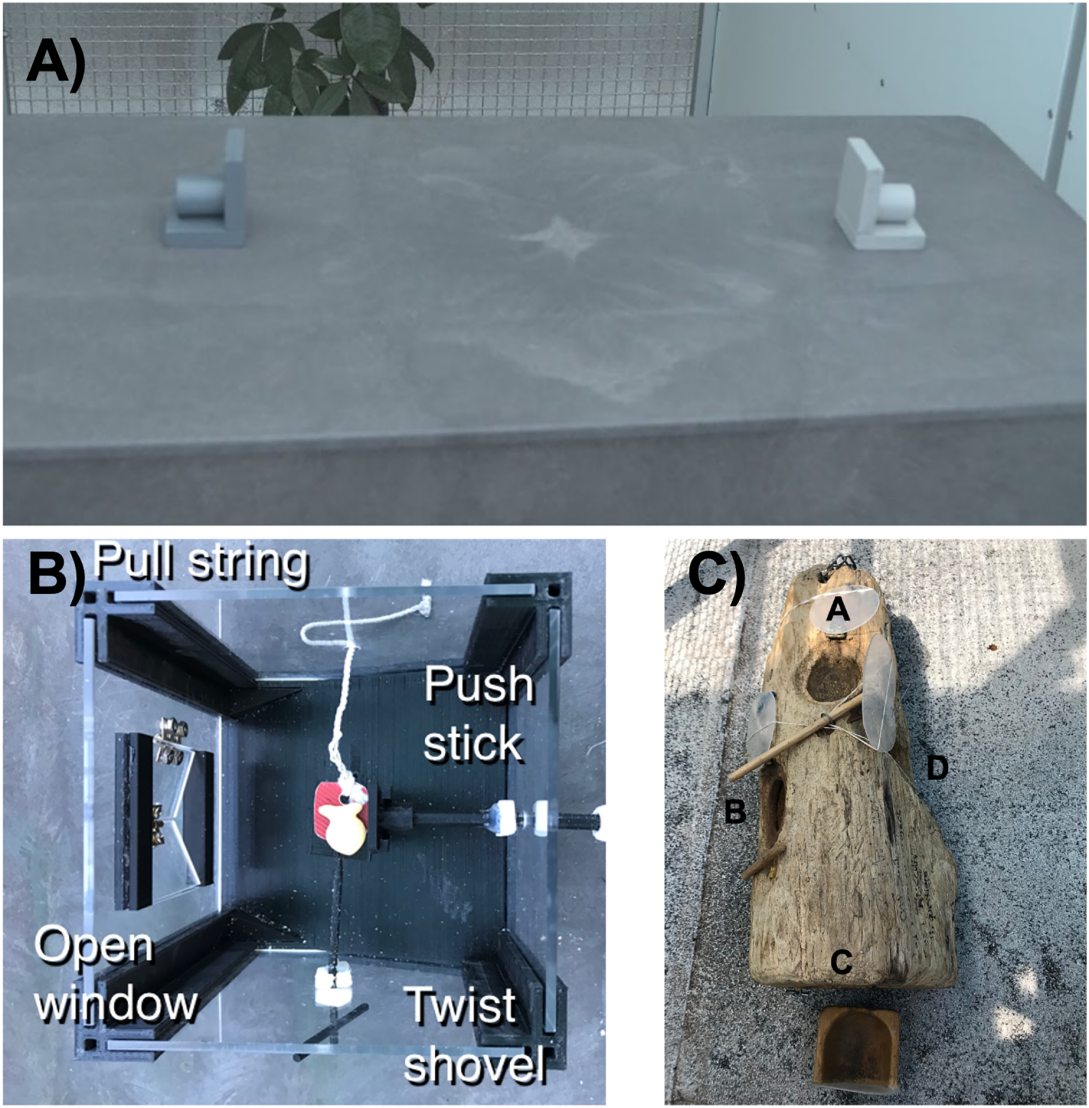
We assessed flexibility as the latency to switch a preference across three contexts illustrated here. (A) We used two colored containers (tubes) in a color reversal learning task, as well as (B) plastic and (C) wooden multi-access boxes that each had four possible ways (loci) to access food. In each context, after a preference for a color/locus was formed, we made the preferred choice non-functional and then measured the latency of the grackle to switch to a new color/locus.

We tested two hypotheses about the construct validity of the reversal learning method as a measure of behavioral flexibility in the great-tailed grackle (*Quiscalus mexicanus*; hereafter “grackle”). First, we determined whether performance on a reversal learning task represents an inherent trait by assessing the repeatability of performance across serial reversals (temporal repeatability). Secondly, we determined whether the inherent trait measured by color reversal learning is likely to represent behavioral flexibility by assessing the cross-contextual repeatability of performance on this task with another task also thought to measure flexibility. Our previous research found that behavioral flexibility does affect innovation ability on a multi-access box (Logan et al., 2023), so here our second hypothesis tested whether individuals show contextual repeatability of flexibility by comparing performance on the color reversal learning task to the latency of solution switching on two different multi-access boxes (Fig. 1). We chose solution switching because it requires similar attention to changing reward contingencies, thus serving as a measure of flexibility, but in a different context (*e.g*., the food is always visible, there is no color association learning required). In other words, in both color reversal learning and solution switching individuals learned a preferred way to obtain food, but then contingencies changed such that food can no longer be obtained with this learned preference and the grackle must be able to switch to a new method. As a human-associated species, the grackle is an ideal subject for this study because the rapid range expansion suggests that they adapted quickly in response to human-induced rapid environmental change (Summers et al., 2023; Wehtje, 2003) and the genus *Quiscalus* has a high rate of foraging innovations in the wild (Grabrucker & Grabrucker, 2010; Lefebvre & Sol, 2008). Therefore, as their environment may select for flexible and innovative behavior, we believe that these tasks are ecologically relevant and will elicit individual variation in performance.

## Methods

### Preregistration details

This experiment was one piece (**H3a** and **H3b**) of a larger project. This project is detailed in the preregistration that was written (2017), submitted to PCI Ecology for peer review (July 2018), and received the first round of peer reviews a few days before data collection began (Sep 2018). We revised and resubmitted this preregistration after data collection had started (Feb 2019) and it passed peer review (Mar 2019) before any of the planned analyses had been conducted. See also the peer review history at PCI Ecology. The hypotheses, methods, and analysis plan for this experiment are described in detail in that peer-reviewed preregistration. We give a summary of these methods here, with any changes from the preregistration summarized in the *Deviations from the preregistration* section in the Supplemental Material and additionally noted in each relevant section of the preregistration (indicated in italics).

### Hypotheses

Our first hypothesis considered whether behavioral flexibility (as measured by reversal learning of a color preference) would be repeatable within individuals across serial reversals. Secondly, we hypothesized that, as an inherent trait, behavioral flexibility results in repeatable performance across other contexts (Fig. 1) that require changing behavior when circumstances change (context 1 = reversal learning on colored tubes, context 2 = plastic multi-access box, context 3 = wooden multi-access box).

### Subjects

Great-tailed grackles were caught in the wild in Tempe, Arizona USA using a variety of trapping methods. All individuals received color leg bands for individual identification and some individuals (*n* = 34) were brought temporarily into aviaries. Grackles were individually housed in an aviary (each 244 cm long by 122 cm wide by 213 cm tall) for a maximum of 6 months where they had *ad lib* access to water at all times. During testing, we removed their maintenance diet for up to 4 h per day. During this time, they had the opportunity to receive high value food items by participating in tests. Individuals were given three to 4 days to habituate to the aviaries before we began testing. At the end of testing, all individuals were released back to the wild at the location where they were caught and no euthanasia of subjects was necessary. This research is carried out in accordance with permits from the US Fish and Wildlife Service (scientific collecting permit number MB76700A-0,1,2), US Geological Survey Bird Banding Laboratory (federal bird banding permit number 23872), Arizona Game and Fish Department (scientific collecting license number SP594338 (2017), SP606267 (2018), and SP639866 (2019)), Institutional Animal Care and Use Committee at Arizona State University (protocol number 17-1594R), and the University of Cambridge ethical review process (non-regulated use of animals in scientific procedures: zoo4/17 (2017)).

### Serial color reversal learning

We first used serial reversal learning to measure grackle behavioral flexibility. Briefly, we trained grackles to search in one of two differently colored containers for food (Fig. 1A). We used a random number generator to select the color (*e.g*., light gray) of the container that would consistently contain a food reward across the initial trials. Within each trial, grackles could choose only one container to look in for food. Eventually, grackles showed a significant preference for the rewarded color container (where preference was defined as a minimum of 17 out of 20 correct choices), completing the initial discrimination trials. We then switched the location of the food to the container of the other color (a reversal). The food reward was then consistently located in the container of this second color (*e.g*., dark gray) across trials until the grackles learned to switch their preference, after which we would again reverse the food to the original colored container (*e.g*., light gray) and so on back and forth until they passed the serial reversal learning experiment passing criterion (formed a preference in two sequential reversals in 50 or fewer trials: Logan et al., 2023). We measured behavioral flexibility on each reversal as the time it took grackles to switch their preference and search in the second colored container on a minimum of 17 out of 20 trials. See the protocol for serial reversal learning here.

### Multi-access boxes

We additionally used two different multi-access boxes (hereafter “MAB”) to assess behavioral flexibility as the latency to switch loci when a preferred locus becomes non-functional. We randomized the order that grackles received each MAB and all grackles were given time to habituate to the MABs prior to testing. We set up the MABs in the aviary of each grackle with food in and around each apparatus in the days prior to testing. At this point all loci were absent or fixed in open, non-functional positions to prevent any early learning of how to solve each apparatus. We began testing when the grackle was eating comfortably from the MAB. For each MAB, the goal was to measure how quickly the grackle could learn to solve each locus, and then how quickly they could switch to attempting to solve a new locus. Consequently, we measured the number of trials to solve a locus and the number of trials until the grackle attempted a new locus after a previously solved locus was made non-functional (solution switching). See protocols for MAB habituation and testing here.

**Plastic multi-access box:** This apparatus consisted of a box with transparent plastic walls (Fig. 1B). There was a pedestal within the box where the food was placed and four different options (loci) set within the walls for accessing the food. One locus was a window that, when opened, allowed the grackle to reach in to grab the food. The second locus was a shovel that the food was placed on such that, when turned, the food fell from the pedestal and rolled out of the box. The third locus was a string attached to a tab that the food was placed on such that, when pulled, the food fell from the pedestal and rolled out of the box. The last locus was a horizontal stick that, when pushed, would shove the food off the pedestal such that it rolled out of the box. Each trial was 10 min long, or until the grackle used a locus to retrieve the food item. We reset the box out of view of the grackle to begin the next trial. To pass criterion for a locus, the grackle had to get food out of the box after touching the locus only once (*i.e*., used a functional behavior to retrieve the food) in more than two trials across two sessions. Afterward, the locus is made non-functional to encourage the grackle to interact with the other loci.

**Wooden multi-access box:** This apparatus consisted of a natural log that contained four compartments (loci) covered by transparent plastic doors (Fig. 1C). Each door opened in a different way (open up like a hatch, out to the side like a car door, pull out like a drawer, or push in). During testing, all doors were closed and food was placed in each locus. Each trial lasted 10 min or until the grack three times, that door was fixed in the open position and the compartment left empty to encourage the grackle to attempt the other loci.

### Repeatability analysis

Repeatability is defined as the proportion of total variation in performance that is attributable to differences among individuals (Nakagawa & Schielzeth, 2010). In other words, performance is likely to represent an inherent trait when there is significant among-individual variation in performance across repeated samples.

To measure repeatability within an individual across serial reversals of a color preference, we modeled the number of trials to pass a reversal (choosing correctly on at least 17 out of 20 sequential trials) as a function of the reversal number (*i.e*., first time the rewarded color is reversed, second time, third time, *etc*.,) and a random effect for individual. The reversal number for each grackle ranged between six to 11 (mean = 7.6) reversals, and the range was based on when individuals were able to pass two sequential reversals in 50 or fewer trials, or (in one case) when we reached the maximum duration that we were permitted to keep grackles in the aviaries and they needed to be released. We thus used the adjusted repeatability (Nakagawa & Schielzeth, 2010) as the variance components for the random effect and residual variance, after accounting for the variance attributed to reversal number, to determine the proportion of variance attributable to differences among individuals. Although our dependent variable (number of trials to reverse) is a count variable, the distribution of values was not appropriate for a poisson regression. When checking the fit of our data to a poisson model, the data were overdispersed and heteroscedastic. However, when log-transformed, the data approximate a normal distribution and are not heteroscedastic, indicating the Gaussian model fits our log-transformed data well.

By design in the serial reversal learning experiment, to reach the experiment ending criteria grackles became faster at switching across serial reversals. We did attempt to run a model that additionally included a random slope to test whether there were consistent individual differences in the rate that grackles switched their preferences across reversals. However, we could not get the model to converge with our sample size and the uninformative priors that were preregistered. We felt most comfortable using the preregistered methods to avoid biasing the model output. To determine the statistical significance of the repeatability value, while accounting for this non-independence of a change in reversal speed over time, we compared the actual performance on the number of trials to switch a preference in each reversal to simulated data where birds performed randomly within each reversal.

We tested for contextual repeatability by modeling the variance in latency (in seconds) to switch a preference among and within individuals across three behavior switching contexts. Note that the time it took to switch a colored tube preference in serial reversal learning was measured in trials, but the time it took to switch loci in the MAB experiment was measured in seconds. We used the trial start times in the serial reversal experiment to convert the latency to switch a preference from number of trials to number of seconds. Therefore, the contexts across which we measured repeatability of performance were the latency to switch a preference to a new color in the color reversal learning task and latency to switch to a new locus after a previously solved locus was made non-functional on both MABs.

We used the DHARMa package (Hartig, 2019) in R to test whether our model fit our data and was not heteroscedastic, zero-inflated or over-dispersed. We used the MCMCglmm package (Hadfield, 2010), with uninformative priors, to model the relationships of interest for our two hypotheses.

### Open data

All data are available at the Knowledge Network for Biocomplexity’s data repository: https://knb.ecoinformatics.org/view/doi:10.5063/F1VX0F0W (McCune et al., 2022).

## Results

Our sample size was nine individual grackles and 68 total data points (one value for each of the 6–11 reversals that each grackle experienced) for our first hypothesis testing temporal repeatability of reversal learning performance.

**Figure 2:**
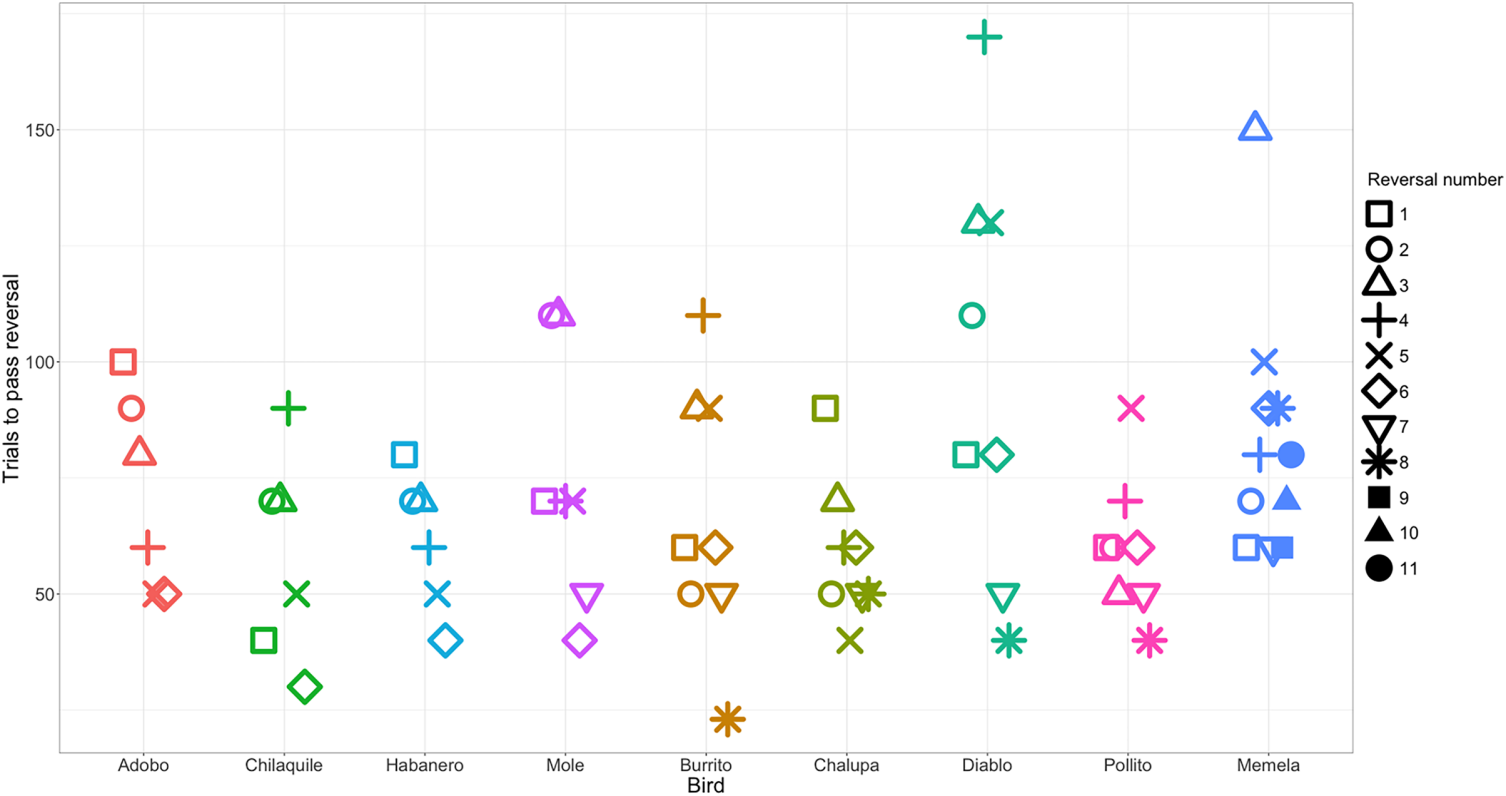
The number of trials each individual took to reverse a preference across serial reversals. The clustering of data points within each individual illustrates the temporal repeatability in performance. Each reversal is indicated by a different shape. Individuals are grouped by color and arranged from fastest to slowest to complete the serial reversal experiment. Note that as per the serial reversal experimental design, data from nearly all individuals include two reversals at or below 50 trials. The one exception was Memela, who never increased the speed to switch her preference.

**Figure 3:**
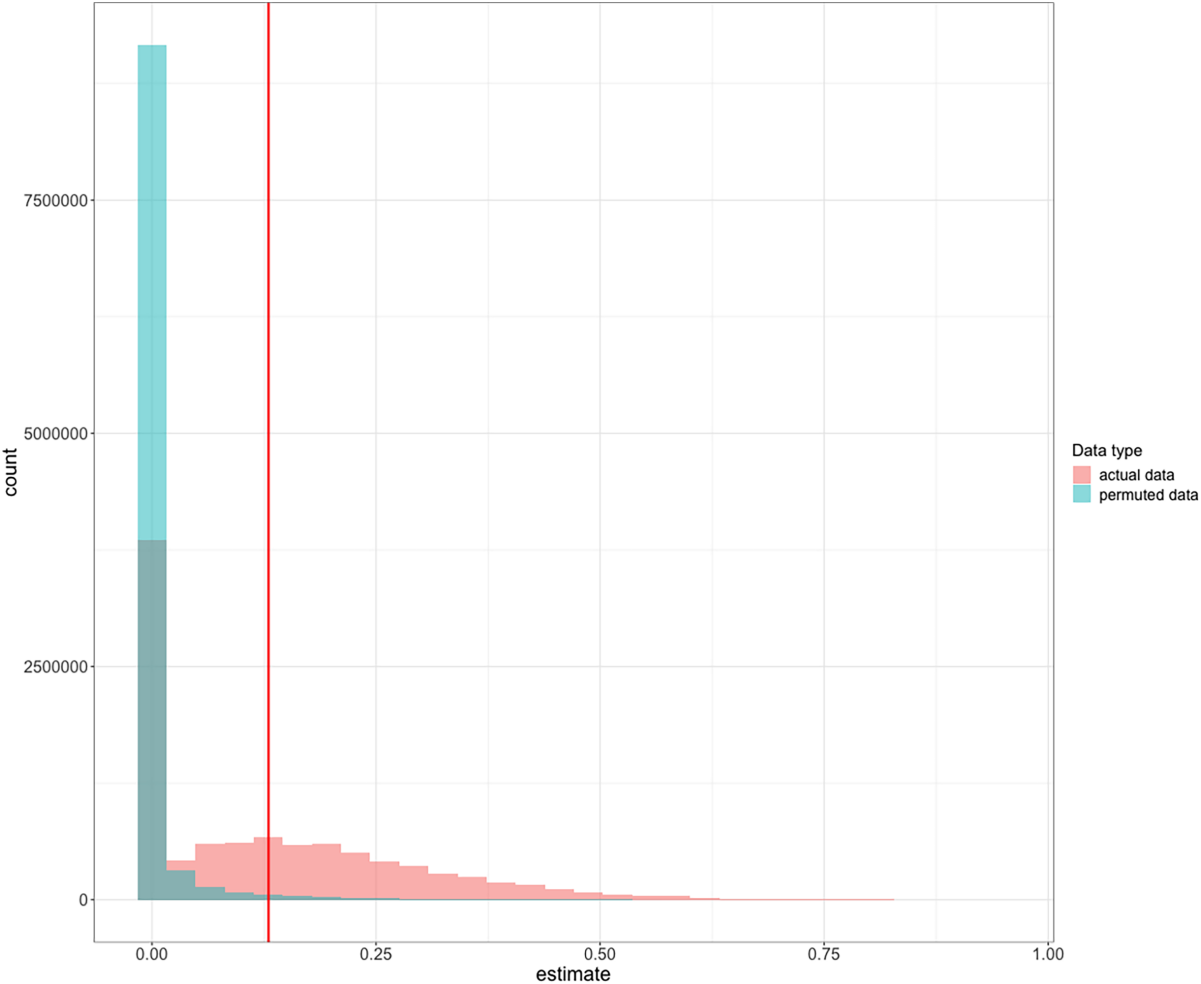
Frequency histograms of posterior repeatability estimates from the model testing the latency to switch a color preference in a serial reversal learning experiment. To determine the significance of our repeatability value while accounting for the non-independence of the serial reversal learning experimental design, we compared our repeatability value to repeatability posterior estimates calculated from permuted data where birds performed randomly within each reversal. Estimates from actual data (red) are compared to the distribution of estimates from randomized permutations of the data (green). The vertical red line at 0.13 is the observed mean repeatability estimate reported in this manuscript and it was significantly greater than random.

Performance was repeatable within individuals within the context of reversal learning (Fig. 2): we obtained a repeatability value of 0.13 (95% credible interval CI [4.64 × 10^−16^– 0.43]). We found that, although the lower bound of the credible interval is approximately zero, the mean repeatability value was significantly greater than expected if birds were performing randomly (*p* = 0.003; Nakagawa & Schielzeth (2010)). Furthermore, the distribution of the posterior estimates for the actual data were much less skewed towards zero compared to the permuted data of birds performing randomly (Fig. 3; see analysis details in the R code for Analysis Plan > P3a), though with the uncertainty we cannot completely exclude that individual identity might not influence performance. Consequently, and as preregistered, we did not conduct the analysis for the P3a alternative to determine whether a lack of repeatability was due to motivation or hunger.

**Figure 4:**
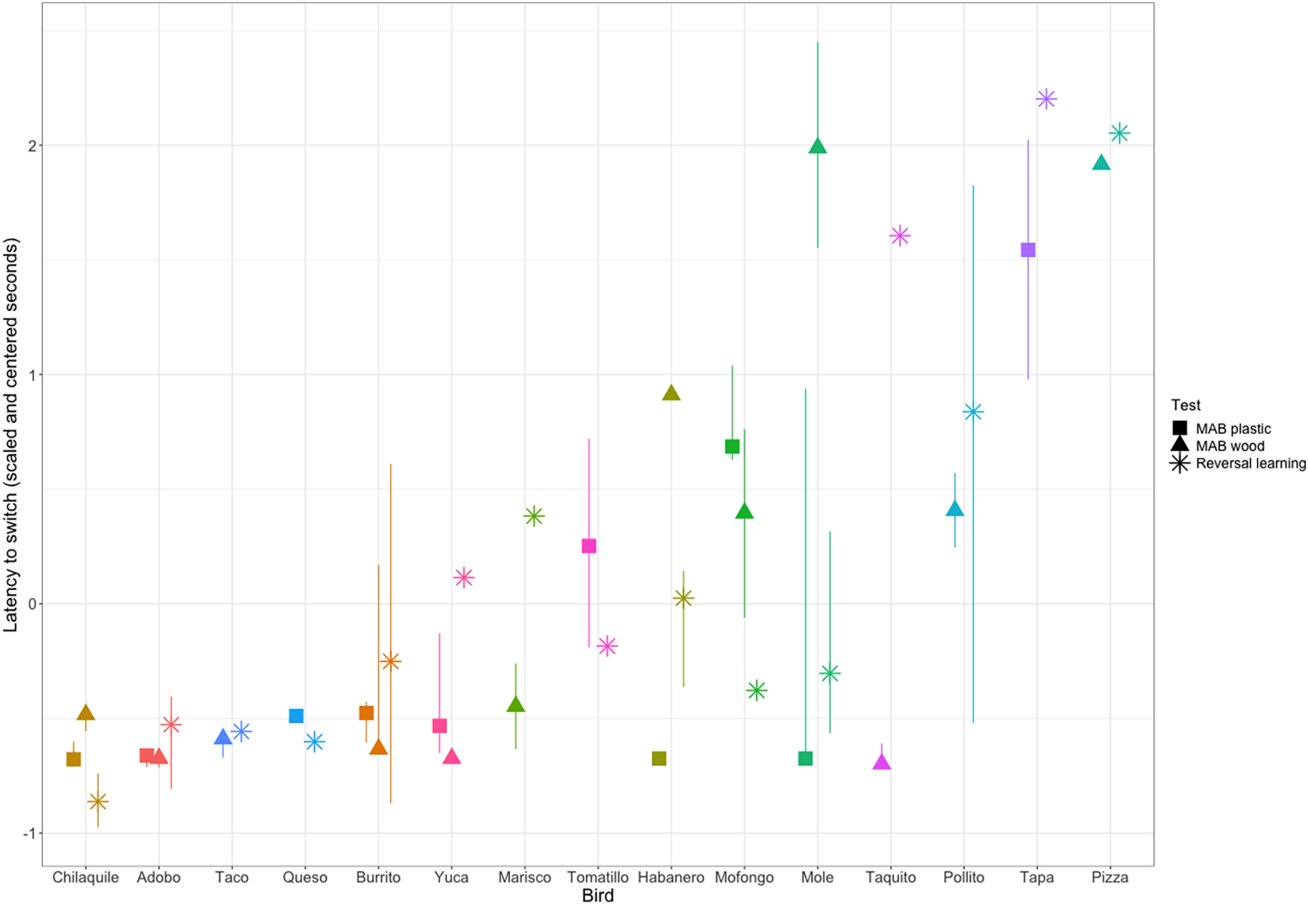
Grackle performance on the different contexts for measuring behavioral flexibility: multi-access box (MAB) plastic (square symbol), MAB wood (triangle symbol), and reversal learning with color tubes (star symbol). Points indicate the (scaled and centered) median performance of an individual in each context, the lines indicate the interquartile range of variation in performance across multiple switches within a context. Some individuals participated in a context, but did not experience multiple preference switches and so there is a point, but no line. Additionally, some individuals are missing points for a given context because they did not participate. Grackles are ordered on the x-axis from fastest (left) to slowest (right).

We then assessed the repeatability of performance across contexts by quantifying whether individuals that were fast to switch a preference in the color reversal task were also fast to switch to attempting a new solution after passing criterion on a different solution on the two MAB tasks. We converted our metric of reversal speed from trials to reverse to seconds to reverse so the measures across contexts would be on the same scale. We had repeated measures across contexts for 15 grackles that participated in at least one color reversal and one solution switch on either or both MAB tasks. We found significant repeatability across contexts (R = 0.36, CI [0.10–0.64], *p* = 0.01; Fig. 4), where latency to switch was consistent within individuals and different among individuals.

## Discussion

We found that individual grackles were consistent in their behavioral flexibility performance during multiple assessments within the same context, and across multiple assessments in different contexts. This indicates that (1) the different methods we used to measure behavioral flexibility all likely measure the same latent inherent aspects affecting performance and (2) there is consistent individual variation in behavioral flexibility, which could impact other traits such as survival and fitness in novel areas, foraging, or social behavior.

In behavioral and cognitive research on animals, it is important to determine that the chosen method measures the trait of interest (construct validity). Many experimental methods may lack construct validity because they were adapted from research on other species (*e.g*., from humans: Wood et al., 1980), applied to new contexts (*e.g*., from captive to wild animals: McCune et al., 2019), or created from an anthropomorphic perspective (*e.g*., mirror self recognition tasks: Kohda et al., 2022). Funding and logistical limitations result in few researchers assessing the appropriateness of their methods by testing construct validity through convergent (similar performance across similar tasks) and discriminant validity (different performance across different tasks). Although our sample size was small, which likely led to moderately large credible intervals, we still found significant temporal and contextual repeatability of switching performance. This evidence for convergent validity indicates these similar tasks are likely assessing aspects of the same latent trait or traits (Morand-Ferron, Reichert & Quinn, 2022; Völter et al., 2018). However, performance can potentially be affected by many traits, so future studies manipulating other factors that might influence performance are needed to continue to pinpoint the latent traits governing aspects of performance on cognitive tasks. Thus, it is important to also test for discriminant validity by comparing performance on flexibility tasks with tasks intended to measure different cognitive abilities. For example, it is possible that performance on serial reversal learning and solution switching on the MAB tasks is reflective of a trait other than behavioral flexibility, like inhibition (MacLean et al., 2014). Indeed, we previously found that the more flexible grackles on the serial reversal learning task were also better able to inhibit responding to a non-rewarded stimulus in a go/no-go task thought to measure self-control (Logan et al., 2021). Consequently, more research is needed to interpret whether some aspect of performance on the go/no-go task reflects behavioral flexibility or whether performance on the reversal learning task is influenced by inhibition.

The repeatability estimate for cross-contextual switching performance was higher than the estimate for switching performance within a context, indicating that a larger portion of the variance in cross-contextual performance is attributable to individual differences (lower residual variance and/or greater among-individual variance). Performance on a task likely depends on multiple cognitive processes, some of which might be more repeatable than others. For example, Lukas et al. (2022) found that performance on the serial reversal learning task was related to two distinct components-the rate of updating an attraction to a colored tube (phi) and the likelihood of deviating from the learned attractions (lambda), where phi appeared to show more individual consistency than lambda. Repeatability might be higher for cross-contextual switching depending on which cognitive processes dominate in a given task and across contexts. Variation in the design of our tasks may lead to higher residual variance in individual performance across reversals because food is hidden in the serial reversal learning task but clearly visible behind transparent plastic barriers in both MAB tasks. After a reversal, to determine which of the two colored tubes to search in for food, grackles cannot rely on short term memory of the previous location of food, they must have some motivation to search in a new color of tube (lambda). Consequently, it is possible that higher within-individual variation in performance across serial reversals in the latency to switch was related to the other factors affecting an individual’s decision-making on each trial, like conflicting memories of reward history for each color tube or a tendency to make a choice based on a side bias. In contrast, in the MAB tasks, even if the previously rewarded option is non-functional the grackles can clearly see that the food is still there and which may facilitate motivation to change their behavior regardless of past memories of reward contingencies or bias towards certain stimuli.

The functional importance of behavioral flexibility is that it is thought to facilitate invasion success by allowing individuals to quickly change their behavior when circumstances change in new environments. For example, flexible grackles may innovate new foraging techniques or generalize standard techniques to new food items in novel areas. The great-tailed grackle has rapidly expanded its range (Summers et al., 2023; Wehtje, 2003), implying that it is able to have high survival and fitness in the face of environmental change. Although grackles are a behaviorally flexible species (Logan, 2016), we show here that there are consistent individual differences among grackles in how quickly they are able to change their behavior when circumstances change in multiple different contexts. While some grackles were consistently faster at changing their behavior (*e.g*., Chilaquile), others were consistently slower (*e.g*., Yuca). This consistency in performance may seem contradictory to our previous research where we found that we are able to manipulate grackles to be more flexible using serial reversal learning (Logan et al., 2023). That behavioral flexibility is both repeatable within individuals across reversals, indicating it is an inherent trait, as well as being manipulatable through serial reversals, aligns with the idea of behavioral reaction norms (Sih, 2013). This idea states that individuals can show consistent individual differences in the baseline or average values of a trait of interest across time or contexts, but the plasticity in the expression of the trait can also consistently vary among individuals. Due to our small sample size, we were not able to explicitly test for behavioral reaction norms, but this is an important next step in understanding consistent individual variation in behavioral flexibility in relation to rapid environmental change. Past experience (developmental or evolutionary) with environmental change influences how plastic the individuals are able to be (Sih, 2013). To understand the implications of this individual variation in performance in this species that has experienced much environmental change during the range expansion, our future research investigates how behavioral flexibility may relate to proximity to the range edge (Logan et al., 2020), and the variety of foraging techniques used in the wild (Logan et al., 2019).

By first validating the experimental methods for behavioral and cognitive traits, such that we are more certain that our tests are measuring the intended trait, we are better able to understand the causes and consequences of species, population, and individual differences. Individual variation in behavioral flexibility has the potential to influence species adaptation and persistence under human-induced rapid environmental change (Sih, 2013). Consequently, we believe the results presented here are a timely addition to the field by demonstrating two potential methods for measuring behavioral flexibility that produced repeatable performance in at least one system.

## Supplemental Information

10.7717/peerj.15773/supp-1 Supplemental Information 1 ARRIVE Author checklist. Line number locations for the basic minimum items to include in an article.

10.7717/peerj.15773/supp-2 Supplemental Information 2 Peer-reviewed preregistration. Additional methodological information from the preregistration such as detailed hypotheses, variables and ability to detect actual effects. Any deviations from the preregistration that we made in the final article are also noted.

## Additional Information and Declarations

### Field Study Permissions

The following information was supplied relating to field study approvals (*i.e*., approving body and any reference numbers): This research is carried out in accordance with permits with the US Fish and Wildlife Service (scientific banding permit MB76700A-0,1,2), US Geological Survey Bird Banding Laboratory (federal bird banding permit number 23872), and the Arizona Game and Fish Department (scientific collecting license number SP594338 (2017), SP606267 (2018), and SP639866 (2019)).

### Data Availability

The following information was supplied regarding data availability: All data and code for analyses are available at the Knowledge Network for Biocomplexity: Kelsey McCune, Aaron Blaisdell, Zoe Johnson-Ulrich, Dieter Lukas, Maggie MacPherson, Benjamin Seitz, August Sevchik, & Corina Logan. (2022). Data: Using repeatability of performance within and across contexts to validate measures of behavioral flexibility. Knowledge Network for Biocomplexity. DOI 10.5063/F1VX0F0W.
