# Supplementary material for: Using repeatability of performance within and across contexts to validate measures of behavioral flexibility
Source: PeerJ. 2023 Aug 17;11:e15773. doi: 10.7717/peerj.15773 (PMC10440059; doi:10.7717/peerj.15773)
Supplement: Supplemental Information 2 — Additional methodological information from the preregistration such as detailed hypotheses, variables and ability to detect actual effects. Any deviations from the preregistration that we made in the final article are also noted. [file peerj-11-15773-s002.pdf]

SUPPLEMENTARY MATERIALS

Deviations from the preregistration

In the middle of data collection

1) We originally planned to use a touchscreen test of serial reversal learning as one of the contexts in this experiment. However, on 10 April 2019 we **discontinued the reversal learning experiment on the touchscreen** because it appears to measure something other than what we intended to test and it requires a huge time investment for each bird (which consequently reduces the number of other tests they are available to participate in). This is not necessarily surprising because this is the first time touchscreen tests have been conducted in this species, and also the first time (to our knowledge) this particular reversal experiment has been conducted on a touchscreen with birds. We based this decision on data from four grackles (2 in the flexibility manipulation group and 2 in the flexibility control group; 3 males and 1 female). All four of these individuals showed highly inconsistent learning curves and required hundreds more trials to form each preference when compared to the performance of these individuals on the colored tube reversal experiment. It appears that there is a confounding variable with the touchscreen such that they are extremely slow to learn a preference as indicated by passing our criterion of 17 correct trials out of the most recent 20. We will not include the data from this experiment when conducting the cross-test comparisons in the Analysis Plan section of the preregistration.

2) 16 April 2019: Because we discontinued the touchscreen reversal learning experiment, we **added an additional but distinct multi-access box** task, which allowed us to continue to measure flexibility across three different experiments. There are two main differences between the first multi-access box, which is made of plastic, and the new multi-access box, which is made of wood. First, the wooden multi-access box is a natural log in which we carved out 4 compartments. As a result, the apparatus and solving options are more comparable to what grackles experience in the wild, though each compartment is covered by a transparent plastic door that requires different behaviors to open. Furthermore, there is only one food item available in the plastic multi-access box and the bird could use any of 4 loci to reach it. In contrast, the wooden multi-access box has a piece of food in each of the 4 separate compartments.

Post data collection, pre-data analysis

3) We completed our simulation to explore the lower boundary of a minimum sample size and determined that **our sample size for the Arizona study site is above the minimum** (see details and code in Ability to detect actual effects; 17 April 2020).

4) We originally planned on testing only **adults** to have a better understanding of what the species is capable of, assuming the abilities we are testing are at their optimal levels in adulthood, and so we could increase our statistical power by eliminating the need to include age as an independent variable in the models. Because the grackles in Arizona were extremely difficult to catch, we ended up testing two juveniles in this experiment. The juveniles' performance on the three tests was similar to the adults, therefore we decided not to add age as an independent variable in the models to avoid reducing our statistical power.

Post data collection, mid-data analysis

5) The distribution of values for the "number of trials to reverse" response variable in the P3a analysis was not a good fit for the Poisson distribution because it was overdispersed and heteroscedastic. We log-transformed the data to approximate a normal distribution and it passed all of the data checks. Therefore, we used a Gaussian distribution for our model, which fits the log-transformed data well. (24 Aug 2021)

6) We realized we mis-specified the model and variables for evaluating cross-contextual repeatability P3b analysis. The dependent variable should be latency to switch to a new preference (we previously listed "number of trials to solve", which is more likely indicative of innovation rather than flexibility). Furthermore, to assess performance across contexts, this dependent variable should be the latency to switch in each of the 3 contexts. Note that the time it took to switch a colored tube preference in serial reversal learning was measured in trials, but the time it took to switch loci in the MAB experiment was measured in seconds. We used the trial start times in the serial reversal experiment to convert the latency to switch a preference from number of trials to number of seconds. In line with this change in the dependent variable, the independent variables are only Context (MAB plastic, MAB wood, reversal learning), and reversal number (the number of times individuals switched a preference when the previously preferred color/locus was made non-functional). Additionally, this dependent variable was heteroscedastic when we used a Poisson model, but passed all data checks when we log-transformed it to use a Gaussian model.

PREREGISTRATION (detailed methods)

HYPOTHESES

H3a: Behavioral flexibility within a context is repeatable within individuals. Repeatability of behavioral flexibility is defined as the number of trials to reverse a color preference being strongly negatively correlated within individuals with the number of reversals.

P3a: Individuals that are faster to reverse a color preference in the first reversal will also be faster to reverse a color preference in the second, etc. reversal due to natural individual variation.

P3a alternative: There is no repeatability in behavioral flexibility within individuals, which could indicate that performance is state dependent (e.g., it depends on their fluctuating motivation, hunger levels, etc.). We will determine whether performance on colored tube reversal learning related to motivation by examining whether the latency to make a choice influenced the results. We will also determine whether performance was related to hunger levels by examining whether the number of minutes since the removal of their maintenance diet from their aviary plus the number of food rewards they received since then influenced the results.

H3b: The consistency of behavioral flexibility in individuals across contexts (context 1=reversal learning on colored tubes, context 2=multi-access boxes, context 3=reversal learning on touchscreen) indicates their ability to generalize across contexts. Individual consistency of behavioral flexibility is defined as the number of trials to reverse a color preference being strongly positively correlated within individuals with the latency to solve new loci on each of the multi-access boxes and with the number of trials to reverse a color preference on a touchscreen (total number of touchscreen reversals = 5 per bird).

If P3a is supported (repeatability of flexibility within individuals)…

P3b: …and flexibility is correlated across contexts, then the more flexible individuals are better at generalizing across contexts.

P3b alternative 1: …and flexibility is not correlated across contexts, then there is something that influences an individual's ability to discount cues in a given context. This could be the individual's reinforcement history (tested in P3a alternative), their reliance on particular learning strategies (one alternative is tested in H4), or their motivation (tested in P3a alternative) to engage with a particular task (e.g., difficulty level of the task).

DEPENDENT VARIABLES *P3a and P3a alternative 1*

Number of trials to reverse a preference. An individual is considered to have a preference if it chose the rewarded option at least 17 out of the most recent 20 trials (with a minimum of 8 or 9 correct choices out

of 10 on the two most recent sets of 10 trials). We use a sliding window to look at the most recent 10 trials for a bird, regardless of when the testing sessions occurred.

P3b: additional analysis: individual consistency in flexibility across contexts + flexibility is correlated across contexts

Number of trials to solve a new locus on the multi-access boxes *NOTE: Jul 2022 we realized this variable is more likely to represent innovation, and we mean to assess flexibility here. Therefore we changed this variable to latency to attempt to switch a preference after the previously rewarded color/locus becomes non-functional.*

INDEPENDENT VARIABLES *P3a: repeatable within individuals within a context*

1) Reversal number
2) ID (random effect because repeated measures on the same individuals)

P3a alternative 1: was the potential lack of repeatability on colored tube reversal learning due to motivation or hunger?

1) Trial number
2) Latency from the beginning of the trial to when they make a choice
3) Minutes since maintenance diet was removed from the aviary
4) Cumulative number of rewards from previous trials on that day
5) ID (random effect because repeated measures on the same individuals)
6) Batch (random effect because repeated measures on the same individuals). Note: batch is a test cohort, consisting of 8 birds being tested simultaneously

P3b: repeatable across contexts

NOTE: Jul 2022 we changed the dependent variable to reflect the general latency to switch a preference (in any of the three tasks) and so IVs 3 (Latency to solve a new locus) & 4 (Number of trials to reverse a preference), below, are redundant. Furthermore, we did not include the touchscreen experiment in this manuscript (previously accounted for with IV 5; see the Deviations section). Therefore, despite being listed here in the preregistration as IVs that we proposed to include in the P3b model, in our post-study manuscript we did not include these IVs in the final model. The IVs instead consisted of: Reversal (switch) number, Context (colored tubes, plastic multi-access box, wooden multi-access box) and ID (random effect because there were repeated measures on the same individuals).

1) Reversal (switch) number
2) Context (colored tubes, plastic multi-access box, wooden multi-access box, touchscreen)
3) Latency to solve a new locus
4) Number of trials to reverse a preference (colored tubes)
5) Number of trials to reverse a preference (touchscreen)
6) ID (random effect because repeated measures on the same individuals)

ANALYSIS PLAN *P3a: repeatable within individuals within a context (reversal learning)*

Analysis: Is reversal learning (colored tubes) repeatable within individuals within a context (reversal learning)? We will obtain repeatability estimates that account for the observed and latent scales, and then compare them with the raw repeatability estimate from the null model. The repeatability estimate indicates how much of the total variance, after accounting for fixed and random effects, is explained by individual differences (ID). We will run this GLMM using the MCMCglmm function in the MCMCglmm package (Hadfield, 2010) with a Poisson distribution and log link using 13,000 iterations with a thinning interval of 10, a burnin of 3,000, and minimal priors [V=1, nu=0; Hadfield (2014)]. We will ensure the GLMM shows acceptable convergence [i.e., lag time autocorrelation values <0.01; Hadfield (2010)], and adjust parameters if necessary.

NOTE (Aug 2021): our data checking process showed that the distribution of values of the data (number of trials to reverse) in this model was not a good fit for the Poisson distribution because it was overdispersed and heteroscedastic. However, when log-transformed the data approximate a normal distribution and pass all of the data checks, therefore we used a Gaussian distribution for our model, which fits the log-transformed data well.

To roughly estimate our ability to detect actual effects (because these power analyses are designed for frequentist statistics, not Bayesian statistics), we ran a power analysis in G*Power with the following settings: test family=F tests, statistical test=linear multiple regression: Fixed model (R^2 deviation from zero), type of power analysis=a priori, alpha error probability=0.05. The number of predictor variables was restricted to only the fixed effects because this test was not designed for mixed models. We reduced the power to 0.70 and increased the effect size until the total sample size in the output matched our projected sample size (n=32). The protocol of the power analysis is here:

Input:

Effect size f^2 = 0.21

err prob = 0.05

Power (1- err prob) = 0.7

Number of predictors = 1

Output:

Noncentrality parameter = 6.7200000

Critical F = 4.1708768

Numerator df = 1

Denominator df = 30

Total sample size = 32

Actual power = 0.7083763

This means that, with our sample size of 32, we have a 71% chance of detecting a medium effect (approximated at f^2=0.15 by Cohen, 1988).

P3a alternative: was the potential lack of repeatability on colored tube reversal learning due to motivation or hunger?

Analysis: Because the independent variables could influence each other or measure the same variable, I will analyze them in a single model: Generalized Linear Mixed Model [GLMM; MCMCglmm function, MCMCglmm package; Hadfield (2010)] with a binomial distribution (called categorical in MCMCglmm) and logit link using 13,000 iterations with a thinning interval of 10, a burnin of 3,000, and minimal priors (V=1, nu=0) (Hadfield, 2014). We will ensure the GLMM shows acceptable convergence [lag time autocorrelation values <0.01; Hadfield (2010)], and adjust parameters if necessary. The contribution of each independent variable will be evaluated using the Estimate in the full model. *NOTE (Apr 2021): This analysis is restricted*

to data from their first reversal because this is the only reversal data that is comparable across the manipulated and control groups.

To roughly estimate our ability to detect actual effects (because these power analyses are designed for frequentist statistics, not Bayesian statistics), we ran a power analysis in G*Power with the following settings: test family=F tests, statistical test=linear multiple regression: Fixed model (R^2 deviation from zero), type of power analysis=a priori, alpha error probability=0.05. We reduced the power to 0.70 and increased the effect size until the total sample size in the output matched our projected sample size (n=32). The number of predictor variables was restricted to only the fixed effects because this test was not designed for mixed models. The protocol of the power analysis is here:

Input:

Effect size f^2 = 0.31

err prob = 0.05

Power (1- err prob) = 0.7

Number of predictors = 4

Output:

Noncentrality parameter = 11.4700000

Critical F = 2.6684369

Numerator df = 4

Denominator df = 32

Total sample size = 37

Actual power = 0.7113216

This means that, with our sample size of 32, we have a 71% chance of detecting a large effect (approximated at f^2=0.35 by Cohen, 1988).

P3b: individual consistency across contexts

Analysis: Do those individuals that are faster to reverse a color preference also have lower latencies to switch to new options on the multi-access box? A Generalized Linear Mixed Model [GLMM; MCMCglmm function, MCMCglmm package; (Hadfield, 2010) will be used with a Poisson distribution and log link using 13,000 iterations with a thinning interval of 10, a burnin of 3,000, and minimal priors (V=1, nu=0) (Hadfield, 2014). We will ensure the GLMM shows acceptable convergence [lag time autocorrelation values <0.01; Hadfield (2010)], and adjust parameters if necessary. We will determine whether an independent variable had an effect or not using the Estimate in the full model.

To roughly estimate our ability to detect actual effects (because these power analyses are designed for frequentist statistics, not Bayesian statistics), we ran a power analysis in G*Power with the following settings: test family=F tests, statistical test=linear multiple regression: Fixed model (R^2 deviation from zero), type of power analysis=a priori, alpha error probability=0.05. We reduced the power to 0.70 and increased the effect size until the total sample size in the output matched our projected sample size (n=32). The number of predictor variables was restricted to only the fixed effects because this test was not designed for mixed models. The protocol of the power analysis is here:

Input:

Effect size f^2 = 0.21

err prob = 0.05

Power (1- err prob) = 0.7

Number of predictors = 1

Output:

Noncentrality parameter = 6.7200000

Critical F = 4.1708768

Numerator df = 1

Denominator df = 30

Total sample size = 32

Actual power = 0.7083763

This means that, with our sample size of 32, we have a 71% chance of detecting a medium effect (approximated at f^2=0.15 by Cohen, 1988).
